# Addition of Five Novel Fungal Flora to the Xylariomycetidae (Sordariomycetes, Ascomycota) in Northern Thailand

**DOI:** 10.3390/jof9111065

**Published:** 2023-10-31

**Authors:** Milan C. Samarakoon, Saisamorn Lumyong, Ishara S. Manawasinghe, Nakarin Suwannarach, Ratchadawan Cheewangkoon

**Affiliations:** 1 Department of Entomology and Plant Pathology, Faculty of Agriculture, Chiang Mai University, Chiang Mai 50200, Thailand; ratchadawan.c@cmu.ac.th; 2 Functional Feed Innovation Center (FuncFeed), Faculty of Agriculture, Chiang Mai University, Chiang Mai 50200, Thailand; 3 Center of Excellence in Microbial Diversity and Sustainable Utilization, Chiang Mai University, Chiang Mai 50200, Thailand; scboi009@gmail.com (S.L.); suwan.462@gmail.com (N.S.); 4 Department of Biology, Faculty of Science, Chiang Mai University, Chiang Mai 50200, Thailand; 5 Academy of Science, The Royal Society of Thailand, Bangkok 10300, Thailand; 6 Innovative Institute for Plant Health, Zhongkai University of Agriculture and Engineering, Guangzhou 510225, China; ishara9017@gmail.com

**Keywords:** diversity, *Muscodor*, *Nigropunctata*, nutritional modes, phylogeny, species, taxonomy, *Xenoanthostomella*

## Abstract

The deviation of conventional fungal niches is an important factor in the implications of hidden fungal diversity and global fungal numbers. The Xylariomycetidae (Sordariomycetes, Ascomycota), which is also referred to as xylarialean taxa, has a wide range of species that demonstrate a high degree of variation in their stromatic characteristics, showing either conspicuous or inconspicuous forms. In this study, samples were collected while focusing on temporal and spatial parameters and substrate characteristics. Based on internal transcribed spacer (*ITS*), 28S large subunit rDNA (*LSU*), RNA polymerase II second largest subunit (*RPB2*), and β-tubulin (*TUB2*) multigene phylogeny and morphology, five new species are introduced as *Muscodor brunneascosporus*, *M*. *lamphunensis* (Xylariaceae), *Nigropunctata hydei*, *N*. *saccata* (*Incertae sedis*), and *Xenoanthostomella parvispora* (Gyrotrichaceae). Plant substrates in the early stages of decay and attached to the host were feasible sample niches, with an emphasis on the collection of inconspicuous, hidden xylarialean species. The appearance of inconspicuous saprobic xylarialean forms during the rainy season may be linked to the change in nutritional mode, from endophytic mode during the dry season to saprobic in the wet. Therefore, it would be fascinating to concentrate future research on how seasonal fluctuations affect nutritional mode shifts, especially in northern Thailand, which would provide the optimal spatial characteristics. In order to establish a comprehensive linkage between endophytic and saprobic modes, it is imperative to have a substantial representation of endophytic isolate sequences resembling inconspicuous xylariaceous fungi within publicly accessible databases.

## 1. Introduction

A fungal stroma is a dense cluster of hyphal cells enveloped by a melanized outer layer of substances. Stromatic structures play a crucial role in the life cycle of various fungi, serving as the site for conidial and ascospore development [1]. The characteristics of the substrate or the processes influencing growth are the primary drivers of stromal diversity [2]. According to in vitro studies on *Dussiella tuberiformis* (Clavicipitaceae, Hypocreales), the development of the stromata is primarily influenced by non-reducing sugars, and the formation of the perithecia may be prompted by nutrition and moisture restriction [3]. Stromata appear to have evolved convergently among ascomycetes and basidiomycetes, which act as a resilient propagule that can withstand harsh environmental conditions [1]. Furthermore, stromata development is important for optimum temperature regulation for viable spores and maintaining the water content of the stromata to be able to spread spores, as well as for insects that need an appropriate temperature for their survival [4]. Importantly, early fungal classifications have been focused on the presence or absence, nature, and interior development of like characters for the delimitation of systematic groups of major ranks [5].

The stromatic characters play a major role in the generic and family level classifications of species [6] in the Xylariomycetidae (referred to as “xylarialean taxa” herein). In certain xylarialean species, there is a specialized character known as the clypeus. It consists of stromatic tissue or melanized hyphae that form above the partially submerged or immersed ascomata, which are shield-shaped structures with variable development [2]. Daranagama et al. [7] focused on those stromatic differences of xylarialean taxa and discussed the taxonomic significance of micro-xylariaceous (with inconspicuous ascomata) and macro-xylariaceous (with conspicuous stromata) genera. Following seven stromatic characters among the xylarialean taxa, Samarakoon et al. [8] performed an ancestral character analysis. The results indicated that xylarialean taxa most likely diverged from inconspicuous forms when they first appeared. Additionally, the majority of the xylarialean taxa’s undiscovered sexual morphs are presumably inconspicuous forms. However, there are limited studies on inconspicuous forms compared to conspicuous forms [7].

Taxonomic studies focusing on inconspicuous xylarialean taxa have been limited, probably due to a lack of fresh collections, and many species were described long ago and have never been recollected, especially from tropical locations [7]. It is interesting to note that the genera transferred to new families were previously accepted but had uncertain morphologies and phylogenies. In contrast to conspicuous stromatic xylarialean taxa, those transferred taxa are morphologically unique in having inconspicuous, immersed ascomata that do not have key stromatic characters for delimiting higher ranks. Still, more than 50 *incertae sedis* genera, mostly inconspicuous forms, demanding fresh collections and taxonomic and phylogenetic studies [8].

Hyde et al. [9] provided an outline for the Sordariomycetes, including the Xylariomycetidae, with three orders, as Amphisphaeriales (17 families), Delonicicolales (2 families), and Xylariales (15 families), based on morpho-molecular studies. In addition, due to a lack of molecular data, the Myelospermataceae is treated as a Xylariomycetidae family *incertae sedis*. Samarakoon et al. [8] revisited the Xylariomycetidae following morphology, internal transcribed spacer (*ITS*), 28S large subunit rDNA (*LSU*), RNA polymerase II second largest subunit (*RPB2*), *β*-tubulin (*TUB2*), and translation elongation factor-1α (*TEF-1α*) phylogeny, divergence time estimation, and ancestral character state reconstruction, including fresh collections from China, Italy, Russia, Thailand, and UK. The divergence time estimations showed that Amphisphaeriales and Xylariales diverged 154 (117–190) MYA, whereas Delonicicolales diverged at 161 (123–197) MYA, with crown ages of 127 (92–165) MYA and 147 (111–184) MYA, respectively [8]. The Xylariomycetidae currently comprises 40 families: Amphisphaeriales (21 families), Delonicicolales (2 families), Xylariales (16 families), and Xylariomycetidae family *incertae sedis* (1 family).

Thailand is part of the Indo-Malayan hub of biodiversity, which is distinguished by a special combination of vascular plants and, as a result, many related species of fungi [10]. Using polyphasic approaches and significant advancements in the hierarchical structure of classification, significant progress has been achieved in our understanding of the fungi in northern Thailand. Since 2017, our research has focused on the taxonomy, phylogeny, evolution, and secondary metabolites of xylarialean taxa in northern Thailand. In the provinces of Chiang Mai, Chiang Rai, Lampang, Nan, Phayao, and Phrae, we have introduced 20 new species and five new genera (*Magnostiolata*, *Melanostictus*, *Neoamphisphaeria*, *Nigropunctata*, and *Paravamsapriya*) until 2022 [8,11,12,13]. Based on our previous studies, there is a high possibility of the addition of new inconspicuous xylarialean species from northern Thailand. In this study, we introduce five new inconspicuous xylarialean species collected from northern Thailand and describe them based on morphology and *ITS*, *LSU*, *RPB2*, and *TUB2* multigene phylogeny.

## 2. Materials and Methods

### 2.1. Collection, Isolation, and Morphological Studies

Fresh specimens were collected mainly during the rainy season in 2022 from northern Thailand. External examinations were carried out using a stereomicroscope (Leica M205 FCA-FA, Leica Microsystems Ltd., Hessian, Germany) and photographed with a Leica DMC6200 digital microscope camera. Microscopic photography was performed using a Nikon DS-Ri2 camera connected with a Nikon ECLIPSE Ni (Tokyo, Japan) compound microscope, as described by Samarakoon et al. [8]. Where necessary, Melzer’s reagent, Congo red, and Indian ink were used. The photographs included in the figures were edited using Adobe Photoshop CS6 (Adobe Systems, San Jose, CA, USA) and measured using the Tarosoft (R) Image Framework (v. 0.9.7).

The herbarium specimens were deposited in the Chiang Mai University Herbarium (CMUB), Sustainable Development of Biological Resources Research Herbarium (SDBR), Center of Microbial Diversity, Sustainable Utilization, Department of Biology, Faculty of Science, Chiang Mai University, the Mae Fah Luang University Herbarium (MFLU), Chiang Rai, Thailand, and the Herbarium of Cryptogams Kunming Institute of Botany Academia Sinica (KUN-HKAS), Chinese Academy of Sciences, Kunming, China. New taxa were linked with the MycoBank (https://www.mycobank.org, accessed on 18 October 2023).

### 2.2. DNA Extraction, PCR Amplification, and Sequencing

DNA extractions were performed directly from the fruiting bodies. Total DNA extraction kits were used according to the manufacturer’s instructions (PureDireX, Genomic DNA Isolation Kit, The BIO-HELIX Co., Ltd., New Taipei City, Taiwan).

The *ITS* (ITS5/ITS4; [14]), *LSU* (LR0R/LR5; [15]), 18S small subunit rDNA (*SSU*) (NS1/NS4; [14]), *RPB2* (fRPB2-5f/fRPB2-7cR; [16]), *TUB2* (T1/T22; [17]), and *TEF-1α* (EF1-983F/EF1-2218R; [18]) gene regions were amplified using the PCR protocols described by Samarakoon et al. [8]. The total volume of 25 μL contained 12.5 μL of 2X Taq Plus Master Mix (Taq DNA Polymerase, dNTP, an optimized buffer system, and Dye Plus) (Nanjing Vazyme Biotech Co., Ltd., Nanjing, China), 1 μL of each primer, 9.5 μL of double-distilled water, and 1 μL (25–50 ng) of DNA template. All of the PCR products were immediately subjected to 4 °C and visualized on 1% agarose electrophoresis gels using a 100 bp + 1.5 kb DNA ladder labeled with stain (0.01% bromophenol blue, 0.25 M EDTA, 50% glycerol) (SibEnzyme, Nowosibirsk, Russia) and SafeView I nuclear staining dye (1 µL/10 mL of agarose). PCR products were sent to Apical Scientific SDN. BHD. (Seri Kembangan, Malaysia) for purification and DNA sequencing.

### 2.3. Phylogenetic Analyses

All of contig sequences were searched in BLASTn (https://www.ncbi.nlm.nih.gov, accessed on 18 July 2023) [19]. Related sequences for newly acquired sequences were downloaded from GenBank based on the BLASTn results and recent publications (Table 1). FFT-NS-2 Tree-based Progressive Method, 20 PAM/k = 2, was used to align individual loci with the scoring matrix for nucleotide sequences and the 1.0 Gap opening penalty settings of MAFFT V.7.036 (http://mafft.cbrc.jp/alignment/server/, accessed on 20 July 2023). Alignments were manually improved as needed in BioEdit v. 7.0 [20]. TrimAl (v.1.0) (gappyout option) was used to trim the *ITS* and *LSU* sequences [21]. Exon regions of *RPB2* and *TUB2* were extracted with reference to *Barrmaelia macrospora* (CBS 142768).

Characters were assessed as unordered and equally weighted. The best evolutionary model for each gene was found using MrModeltest 2.3 and the Akaike Information Criterion (AIC). Phylogenetic trees were constructed using single and merged alignments of the genetic markers *ITS*, *LSU*, *RPB2*, and *TUB2*. Both maximum likelihood (ML) and Bayesian Inference (BI) methods were employed. The newly obtained sequences have been archived in GenBank for future research reference (Table 1). ML analyses were conducted using RAxMLGUI v.1.3 [22], employing the ML+rapid bootstrap configuration with 1000 replicates. The Bayesian tree was constructed using MCMC sampling within MrBayes v3.1.2 [23,24], comprising 1,000,000 MCMC generations utilizing four chains and partition analysis with 100 sampling frequencies. The initial 2500 trees (25% of the total) were designated as the burn-in phase and were subsequently excluded from the analysis. Posterior probabilities (PP) were calculated using the remaining 7500 trees. The generated trees were visualized using the FigTree v.1.4.0 program [25], and the final figures were crafted using Adobe Illustrator^®^ CS5 (Version 15.0.0, Adobe Systems, San Jose, CA, USA).

**Table 1:** Names, codes, and corresponding GenBank accession numbers of the taxa used in the phylogenetic analyses of this study.

Taxa	Original Code	GenBank Accession Numbers				References
		*ITS*	*LSU*	*RPB2*	*TUB2*	
*Achaetomium macrosporum*	CBS 532.94	KX976574	KX976699	KX976797	KX976915	[26]
*Alloanthostomella rubicola*	MFLUCC 16-0479 ^T^	KX533455	KX533456	N/A	N/A	[27]
*Amphirosellinia nigrospora*	HAS T 91092308 ^T^	GU322457	N/A	GQ848340	GQ495951	[28]
*Anthostomella formosa*	MFLUCC 14-0170	MW240652	MW240582	N/A	MW820917	[8]
*A*. *helicofissa*	MFLUCC 14-0173 ^T^	MW240653	MW240583	KP340534	KP406617	[8,29]
*A*. *lamiacearum*	MFLU 18-0101 ^T^	MW240669	MW240599	MW658648	N/A	[8]
*A*. *obesa*	MFLUCC 14-0171 ^T^	KP297405	KP340546	KP340533	N/A	[29]
*Anthostomelloides krabiensis*	MFLUCC 15-0678 ^T^	KX305927	KX305928	KX305929	N/A	[30]
*Astrocystis sublimbata*	HAS T 89032207	GU322447	N/A	GQ844834	GQ495940	[28]
*Barrmaelia macrospora*	CBS 142768 ^T^	KC774566	KC774566	MF488995	MF489014	[31,32]
*B*. *moravica*	CBS 142769 ^T^	MF488987	MF488987	MF488996	MF489015	[32]
*B*. *rappazii*	CBS 142771 ^T^	MF488989	MF488989	MF488998	MF489017	[32]
*Biscogniauxia nummularia*	MUCL 51395 ^T^	KY610382	KY610427	KY624236	KX271241	[33]
*Brunneiperidium involucratum*	MFLUCC 14-0009 ^T^	KP297399	KP340541	KP340527	KP406610	[29]
*Camillea obularia*	A TCC 28093	KY610384	KY610429	KY624238	KX271243	[33]
*Chaetomium elatum*	CBS 374.66	KC109758	KC109758	KF001820	KC109776	[34]
*Circinotrichum circinatum*	CBS 148326	ON400743	ON400796	ON399328	N/A	[35]
*C*. *maculiforme*	CBS 140016 ^T^	KR611874	KR611895	ON399338	N/A	[35]
*Clypeosphaeria mamillana*	CBS 140735 ^T^	KT949897	KT949897	MF489001	MH704637	[32,36,37]
*Collodiscula leigongshanensis*	GZUH 0107 ^T^	KP054281	KP054282	KR002588	KR002587	[38]
*Coniocessia maxima*	CBS 593.74 ^T^	GU553332	MH878275	N/A	N/A	[39,40]
*C*. *nodulisporioides*	CBS 281.77 ^T^	MH861061	MH872831	N/A	N/A	[40]
*Creosphaeria sassafras*	S TMA 14087	KY610411	KY610468	KY624265	KX271258	[33]
*Digitodochium amoenum*	CBS 147285 ^T^	ON869303	ON869303	ON808481	ON808525	[41]
*Emarcea castanopsidicola*	CBS 117105 ^T^	AY603496	MK762717	MK791285	MK776962	[12,42]
*E*. *eucalyptigena*	CBS 139908 ^T^	KR476733	MK762718	MK791286	MK776963	[12,43]
*Entalbostroma erumpens*	ICMP 21152 ^T^	KX258206	N/A	KX258204	KX258205	[44]
*Entoleuca mammata*	J.D.R. 100	GU300072	N/A	GQ844782	GQ470230	[28]
*Entosordaria perfidiosa*	CBS 142773 ^T^	MF488993	MF488993	MF489003	MF489021	[32]
*E*. *quercina*	CBS 142774 ^T^	MF488994	MF488994	MF489004	MF489022	[32]
*Graphostroma platystomum*	CBS 270.87 ^T^	JX658535	DQ836906	KY624296	HG934108	[33,45,46,47]
*Gyrothrix encephalarti*	CBS 146684 ^T^	MT373376	MT373358	ON399342	N/A	[35,48]
*G. eucalypti*	CBS 146023 ^T^	MN562109	MN567617	ON399346	N/A	[35,49]
*G*. *podosperma*	MFLUCC 16-0243 ^T^	KX505957	KX505958	KX789496	KX789495	[27]
*G*. *podosperma*	CBS 148804	ON400756	ON400810	ON399343	N/A	[35]
*G*. *verticillata*	CBS 148806	ON400759	ON400813	ON399318	N/A	[35]
*Hansfordia pruni*	CBS 194.56 ^T^	MK442585	MH869122	KU684307	N/A	[40,50,51]
*H*. *pulvinata*	CBS 144422	MK442587	MK442527	N/A	N/A	[50]
*Hypocreodendron sanguineum*	J.D.R. 169 ^T^	GU322433	N/A	GQ844819	GQ487710	[28]
*Induratia apiospora*	A TCC 60639 ^T^	OP862879	OP862881	OP879469	OP879468	[52]
*Kretzschmaria deusta*	CBS 163.93	KC477237	KY610458	KY624227	KX271251	[33,53]
*K*. *guyanensis*	HAS T 89062903	GU300079	N/A	GQ844792	GQ478214	[28]
*Kretzschmariella culmorum*	J.D.R. 88	KX430043	N/A	KX430045	KX430046	[44]
*Linosporopsis ischnotheca*	CBS 145761 ^T^	MN818952	MN818952	MN820708	MN820715	[54]
*L*. *ochracea*	CBS 145999 ^T^	MN818958	MN818958	MN820714	MN820721	[54]
*Lopadostoma dryophilum*	CBS 133213 ^T^	KC774570	KC774570	KC774526	MF489023	[31,32]
*L*. *quercicola*	CBS 133212 ^T^	KC774610	KC774610	KC774558	N/A	[31]
*L*. *turgidum*	CBS 133207 ^T^	KC774618	KC774618	KC774563	MF489024	[31]
*Magnostiolata mucida*	MFLU 19-2133 ^T^	MW240673	MW240603	MW658652	MW775618	[8]
*Melanographium phoenicis*	MFLUCC 18-1481 ^T^	MN482677	MN482678	N/A	N/A	[55]
*M*. *smilacis*	MFLU 21-0075 ^T^	MZ538514	MZ538548	N/A	N/A	[56]
*Microdochium lycopodinum*	CBS 125585 ^T^	JF440979	JF440979	KP859125	KP859080	[57,58]
*M*. *phragmitis*	CBS 285.71 ^T^	KP859013	KP858949	KP859122	KP859077	[58]
*Muscodor albus*	9_6	HM034857	HM034865	N/A	HM034844	[59]
*M*. *albus*	MON T 620 ^T^	AF324336	N/A	N/A	N/A	[60]
*M*. *brasiliensis*	LGMF1255	KY924493	N/A	N/A	N/A	[61]
*M*. *brasiliensis*	LGMF1256 ^T^	KY924494	N/A	N/A	N/A	[61]
*M*. *brunneascosporus*	CMUB 40020 ^T^	OR507145	OR507158	OR504420	OR519978	This study
*M*. *brunneascosporus*	MFLU 23-0406	OR507146	OR507159	N/A	N/A	This study
*M*. *camphorae*	NFCCI 3236 ^T^	KC481681	N/A	N/A	N/A	[62]
*M*. *cinnanomi*	BCC 38842 ^T^	GQ848369	N/A	N/A	N/A	[63]
*M*. *coffeanum*	COAD 1842 ^T^	KM514680	N/A	KP862881	N/A	[64]
*M*. *coffeanum*	MFLUCC 13-0159	MK634693	MK634694	MK644942	MK644943	[65]
*M*. *coffeanum*	COAD 1900	KP862879	N/A	KP862880	N/A	[64]
*M*. *coffeanum*	CMUB 40022	OR507147	OR507160	N/A	N/A	This study
*M*. *crispans*	MON T 2347 ^T^	EU195297	N/A	N/A	N/A	[66]
*M*. *equiseti*	JCM 18233 ^T^	JX089322	N/A	N/A	N/A	[67]
*M*. *fengyangensis*	CGMCC 2863	HM034855	HM034861	HM034851	HM034842	[59]
*M*. *fengyangensis*	CGMCC 2862 ^T^	HM034856	HM034859	HM034849	HM034843	[59]
*M*. *ghoomensis*	NFCCI 3234 ^T^	KF537625	N/A	N/A	N/A	[68]
*M*. *kashayum*	NFCCI 2947 ^T^	KC481680	N/A	N/A	N/A	[69]
*M*. *lamphunensis*	CMUB 40021 ^T^	OR507148	OR507161	OR504421	OR519979	This study
*M*. *lamphunensis*	MFLU 23-0408	OR507149	OR507162	N/A	N/A	This study
*M*. *musae*	JCM 18230 ^T^	JX089323	N/A	N/A	N/A	[67]
*M*. *oryzae*	JCM 18231 ^T^	JX089321	N/A	N/A	N/A	[67]
*M*. *roseus*	MON T 2098 ^T^	AH010859	N/A	N/A	N/A	[70]
*Muscodor* sp.	SMH 1255	MN250031	AY780069	N/A	AY780119	[12,71]
*M*. *strobelii*	NFCCI 2907 ^T^	JQ409999	N/A	N/A	N/A	[72]
*M*. *suthepensis*	JCM 18232 ^T^	JN558830	N/A	N/A	N/A	[67]
*M*. *sutura*	MSUB 2380 ^T^	JF938595	N/A	N/A	N/A	[73]
*M*. *thailandica*	HKAS 102323	MK762708	MK762715	MK791284	MK776961	[12]
*M*. *thailandica*	MFLUCC 17-2669 ^T^	MK762707	MK762714	MK791283	MK776960	[12]
*M*. *tigerensis*	NFCCI 3172 ^T^	JQ409998	N/A	N/A	N/A	[74]
*M*. *vitigenus*	MON T P-15 ^T^	AY100022	N/A	N/A	N/A	[75]
*M*. *vitigenus*	CE-QCA-O1100	KC771512	N/A	N/A	N/A	Unpublished
*M*. *yucatanensis*	MEXU 25511 ^T^	FJ917287	N/A	N/A	N/A	[76]
*M*. *yucatanensis*	CDA744	KU094056	N/A	N/A	N/A	Unpublished
*M*. *yunnanensis*	CGMCC 3.18908 ^T^	MG866046	MG866038	MG866059	MG866066	[77]
*M*. *ziziphi*	MFLUCC 17-2662 ^T^	MK762705	MK762712	MK791281	MK776958	[12]
*M*. *ziziphi*	HKAS 102300	MK762706	MK762713	MK791282	MK776959	[12]
*Nemania abortiva*	BISH 467 ^T^	GU292816	N/A	GQ844768	GQ470219	[28]
*N*. *macrocarpa*	WSP 265 ^T^	GU292823	MH874423	GQ844776	GQ470226	[28,40]
*N*. *primolutea*	HAS T 91102001 ^T^	EF026121	N/A	GQ844767	EF025607	[28]
*Neoanthostomella bambusicola*	MFLU 18-0796 ^T^	MW240657	MW240587	MW658641	MW775610	[8]
*N*. *fici*	MFLU 19-2765 ^T^	MW114390	MW114445	MW177711	N/A	[78]
*N*. *pseudostromatica*	MFLU 15-1190 ^T^	KU940158	KU863146	N/A	N/A	[79]
*Neogyrothrix oleae*	CBS 146068	MN562137	MN567644	N/A	N/A	[49]
*N*. *oleae*	CBS 146069 ^T^	MN562136	MN567643	N/A	N/A	[49]
*Nigropunctata bambusicola*	MFLU 19-2134	MW240662	MW240592	MW658644	N/A	[8]
*N*. *bambusicola*	MFLU 19-2145 ^T^	MW240664	MW240594	MW658646	N/A	[8]
*N*. *hydei*	CMUB 40018 ^T^	OR507150	OR507163	OR504422	N/A	This study
*N*. *hydei*	MFLU 23-0410	OR507151	OR507164	N/A	N/A	This study
*N*. *nigrocircularis*	MFLU 19-2130 ^T^	MW240661	MW240591	N/A	MW775612	[8]
*N*. *saccata*	MFLU 19-2144 ^T^	MW240663	MW240593	MW658645	MW775613	This study
*N*. *saccata*	MFLU 18-0804	MW240658	MW240588	MW658642	MW775611	This study
*Nigropunctata* sp.	HKAS 122747	OQ158966	OQ170888	N/A	N/A	Unpublished
*N*. *thailandica*	MFLU 19-2118 ^T^	MW240659	MW240589	MW658643	N/A	[8]
*N*. *thailandica*	HKAS 106975	MW240660	MW240590	N/A	N/A	[8]
*Occultitheca rosae*	HKAS 102393 ^T^	MW240672	MW240602	MW658651	MW775617	[8]
*Paraxylaria rosacearum*	TASM 6132 ^T^	MG828941	MG829050	N/A	N/A	[80]
*Peglionia verticiclada*	CBS 127654 ^T^	ON400763	ON400815	ON399352	N/A	[35]
*Pirozynkiomyces brasiliensis*	CBS 112314 ^T^	ON400767	ON400819	ON399341	N/A	[35]
*Podosordaria mexicana*	WSP 176	GU324762	N/A	GQ853039	GQ844840	[28]
*Poronia punctata*	CBS 656.78 ^T^	KT281904	KY610496	KY624278	KX271281	[33,81]
*Pseudoanthostomella delitescens*	MFLUCC 16-0477	KX533451	KX533452	KX789491	KX789490	[27]
*P. pini-nigrae*	MFLUCC 16-0478 ^T^	KX533453	KX533454	KX789492	N/A	[27]
*Pseudoceratocladium polysetosum*	FMR 10750 ^T^	KY853430	KY853490	ON399348	N/A	[35,82]
*P*. *polysetosum*	CBS 126092	MH864077	MH875534	ON399347	N/A	[35,40]
*Pseudocircinotrichum papakurae*	CBS 101373	KR611876	KR611897	N/A	N/A	[35]
*P*. *papakurae*	CBS 140221	ON400768	ON400820	ON399349	N/A	[35]
*Rosellinia buxi*	J.D.R. 99	GU300070	N/A	GQ844780	GQ470228	[28]
*R*. *necatrix*	HAS T 89062904	EF026117	KF719204	GQ844779	EF025603	[28]
*Sarcoxylon compunctum*	CBS 359.61	KT281903	KT281898	KY624230	KX271255	[33,81]
*Selenodriella brasiliana*	CBS 140227 ^T^	ON400769	ON400821	ON399356	N/A	[35]
*S*. *cubensis*	CBS 683.96 ^T^	KP859053	KP858990	N/A	N/A	[58]
*Spiririma gaudefroyi*	CBS 147284 ^T^	ON869320	ON869320	ON808497	ON808541	[41]
*Sordaria fimicola*	CBS 723.96	MH862606	MH874231	DQ368647	N/A	[40,83]
*Stilbohypoxylon elaeicola*	HAS T 94082615	GU322440	N/A	GQ844827	GQ495933	[28]
*Xenoanthostomella calami*	MFLUCC 14-0617A ^T^	ON650684	ON650706	N/A	ON745964	[84]
*X*. *calami*	MFLUCC 14-0617B	ON650685	ON650707	N/A	ON745965	[84]
*X*. *chromolaenae*	MFLUCC 17-1484 ^T^	MN638863	MN638848	MN648729	N/A	[55]
*X*. *chromolaenae*	CBS 148702	ON400784	ON400841	N/A	N/A	[35]
*X*. *chromolaenae*	MFLU 18-0840	MW240668	MW240598	N/A	N/A	[8]
*X*. *cycadis*	CBS 137969 ^T^	KJ869121	KJ869178	ON399350	N/A	[35]
*X*. *cycadis*	CPC 25749	ON400786	ON400843	N/A	N/A	[35]
*X*. *olivacea*	CBS 101185	ON400787	ON400844	ON399351	N/A	[35]
*X*. *parvispora*	CMUB 40019 ^T^	OR507143	OR507156	OR504419	OR519977	This study
*X*. *parvispora*	MFLU 23-0409	OR507144	OR507157	N/A	N/A	This study
*Xenoanthostomella* sp.	MFLUCC 23-0098	OR438208	OR438209	N/A	N/A	Unpublished
*Xenoanthostomella* sp.	MFLUCC 23-182	OR438210	N/A	N/A	N/A	Unpublished
*Xylaria adscendens*	J.D.R. 865	GU322432	N/A	GQ844818	GQ487709	[28]
*X*. *arbuscula*	CBS 126415	KY610394	KY610463	KY624287	KX271257	[33]
*X*. *bambusicola*	WSP 205 ^T^	EF026123	N/A	GQ844802	AY951762	[28]
*X*. *cubensis*	J.D.R. 860	GU991523	N/A	GQ848365	GQ502700	[28]
*X*. *discolor*	HAS T 131023 ^T^	JQ087405	N/A	JQ087411	JQ087414	[28]
*X*. *hypoxylon*	CBS 122620 ^T^	AM993141	KM186301	KM186302	KM186300	[29,85]

Abbreviations: ATCC: American Type Culture Collection, Manassas, VA, USA; BCC: BIOTEC Culture Collection, National Center for Genetic Engineering and Biotechnology, Khlong Luang, Thailand; BISH: Bishop Museum, Honolulu, HI, USA; CBS: Westerdijk Fungal Biodiversity Institute, Utrecht, the Netherlands; CDA: Fitopatologia, Universidade de Brasilia, Campus Universitario Darcy Ribeiro, Asa Norte, Brasilia, Distrito Federal 36570000, Brazil; CE-QCA: The collection of endophytes of the Pontificia Universidad Catolica del Ecuador, Ecuador; CGMCC: China General Microbiological Culture Collection Center, Beijing, China; COAD: Otávio de Almeida Drumond Culture Collection, Universidade Federal de Viçosa, Brazil; CPC: Culture collection of Pedro Crous, housed at CBS; FMR: Culture collection of the Faculty of Medicine at the Rovira i Virgili University, Reus, Spain; GZUH: Guizhou University, Guiyang, China; HAST: Academia Sinica, Taipei, Taiwan; HKAS: Herbarium of Cryptogams Kunming Institute of Botany Academia Sinica, China; ICMP: International Collection of Microorganisms from Plants, Auckland, New Zealand; J.D.R.: Jack D. Rogers, Washington State University, Pullman, WA, USA; JCM: Japan Collection of Microorganisms, Japan; LGMF: LabGeM Culture Collection, Federal University of Parana (UFPR), Curitiba, Brazil; MEXU: Instituto de Biología, Universidad Nacional Autónoma de México, Mexico; MFLU, MFLUCC: Mae Fah Luang University, Chiang Rai, Thailand; MONT: Montana State University Herbarium, Plant Sciences and Plant Pathology, Montana State University, Bozeman, MT, USA; MSUB: Mycological collection of Montana State University, Bozeman, MT, USA; MUCL: Université Catholique de Louvain, Louvain-la-Neuve, Belgium; NFCCI: National Fungal Culture Collection of India (NFCCI), India; SMH: S.M. Huhndorf; STMA: Marc Stadler, Helmholtz-Zentrum für Infektionsforschung, Braunschweig, Germany; TASM: Tashkent Mycological Herbarium of the Institute of Botany, Uzbekistan; WSP: Washington State University, Pullman, WA, USA. Type, authentic and reference collections are denoted in “^T^”; N/A not available.

## 3. Results

### 3.1. Phylogenetic Analyses

**Figure 1:**
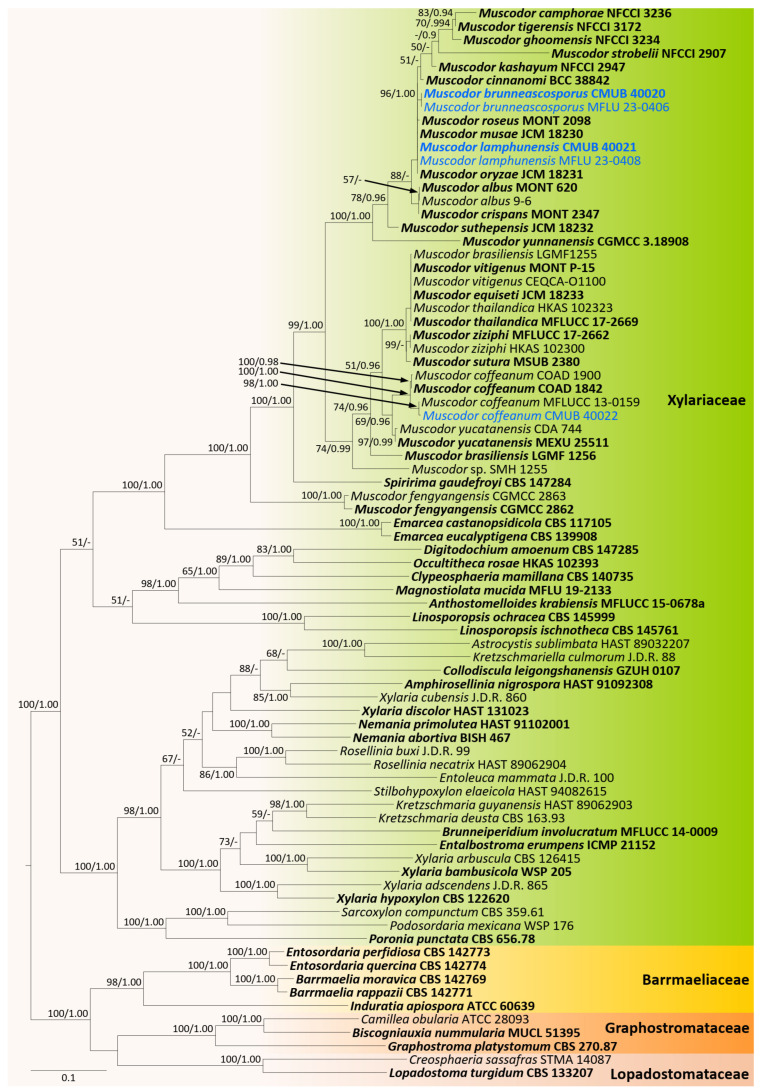
Phylogram generated from maximum likelihood analysis based on combined *ITS*, *LSU*, *RPB2*, and *TUB2* sequence data. The tree is rooted to taxa from the Barrmaeliaceae, Graphostromataceae, and Lopadostomataceae. Bootstrap support values for ML equal to or greater than 50%, PP equal to or greater than 0.9 (ML/PP) are given above or below the nodes. The newly generated sequences are in blue. Type collections are in bold.

**Figure 2:**
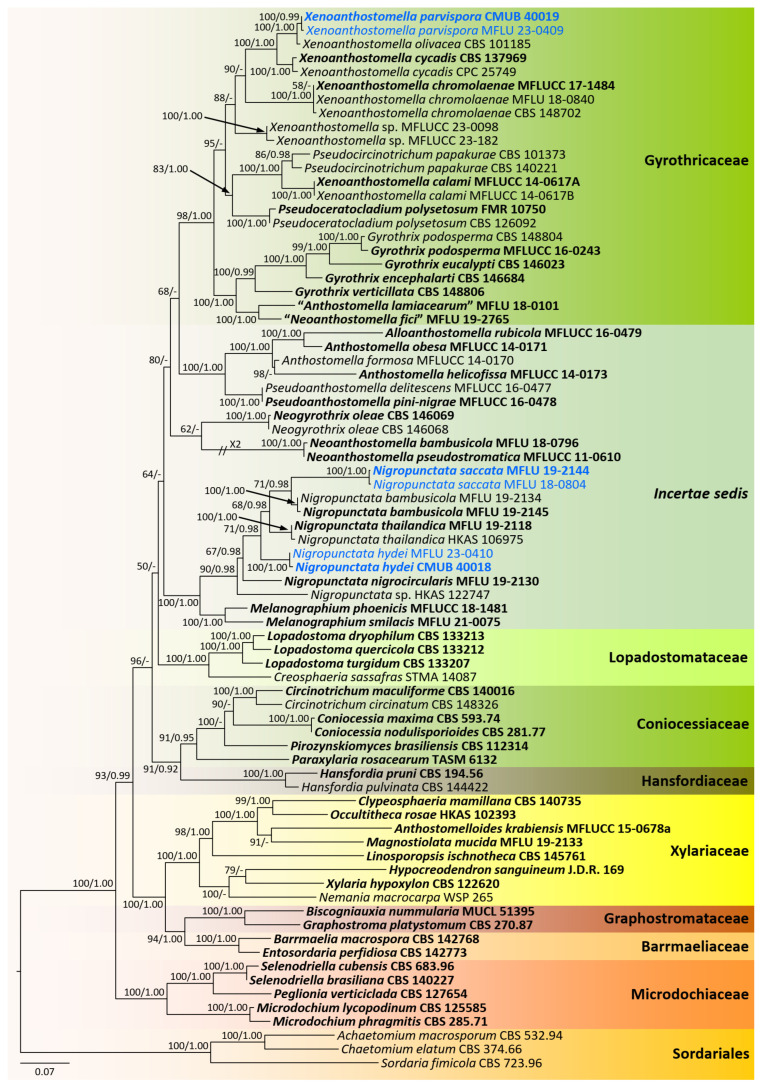
Phylogram generated from maximum likelihood analysis based on combined *ITS*, *LSU*, *RPB2*, and *TUB2* sequence data. The tree is rooted to the taxa belonging to the Sordariales. Bootstrap support values for ML equal to or greater than 50%, PP equal to or greater than 0.9 (ML/PP) are given above or below the nodes. The newly generated sequences are in blue. Type collections are in bold.

Based on the preliminary BLASTn search and morphological studies, new collections were identified into two distinct groups. Therefore, two phylogenetic analyses were conducted as core Xylariaceae and Xylariales genera *incertae sedis* for better resolution of the phylogenetic affinities. The overall tree topology for each analysis was consistent. All the gene regions resulted in the GTR + I + G model. The RAxML analyses of the combined *ITS*, *LSU*, *RPB2*, and *TUB2* datasets yielded the best-scoring trees (Figure 1 and Figure 2). Bayesian posterior probabilities from MCMC were evaluated when the final average standard deviation of split frequencies was less than 0.01.

Analysis 1: The combined sequence alignment comprised 80 strains with 3704 characters, including gaps (*ITS*: 1–737, *LSU*: 738–1578, *RPB2*: 1579–2665, *TUB2*: 2666–3704). Single gene analyses were also performed, and topology and clade stability were compared from combined gene analyses. Tree topology from ML analysis was similar to BI analysis. The best scoring RAxML tree with a final likelihood value of −44754.1890 is presented in Figure 1. The matrix had 2133 (=57.5864% of all sites) constants or ambiguous constants, 1280 parsimony informative sites, and 1862 distinct site patterns. Estimated base frequencies were as follows: A = 0.2057, C = 0.3148, G = 0.2654, T = 0.2141; substitution rates: AC = 1.3568, AG = 4.5921, AT = 1.6135, CG = 0.9632, CT = 7.3071, GT = 1.000; gamma distribution shape parameter α = 0.492847. The completed alignment and tree were archived in TreeBASE under the submission ID: 30765.

Analysis 2: The combined sequence alignment comprised 77 strains with 3612 characters, including gaps (*ITS*: 1–745, *LSU*: 746–1629, *RPB2*: 1630–2673, *TUB2*: 2674–3612). Single gene analyses were also performed, and topology and clade stability were compared from combined gene analyses. Tree topology from ML analysis was similar to that from BI analysis. The best scoring RAxML tree with a final likelihood value of −44146.7002 is presented in Figure 2. The matrix had 1889 (52.2979% of all sites) constants or ambiguous constants, 1334 parsimony informative sites, and 2113 distinct site patterns. Estimated base frequencies were as follows: A = 0.21, C = 0.3068, G = 0.2629, T = 0.2203; substitution rates: AC = 1.1403, AG = 3.2723, AT = 1.5904, CG = 0.8353, CT = 5.4212, GT = 1.000; gamma distribution shape parameter α = 0.7252. The completed alignment and tree were archived in TreeBASE under the submission ID: 30764.

CMUB 40020, CMUB 40021, CMUB 40022, MFLU 23-0406, and MFLU 23-0408 cluster with *Muscodor* in the Xylariaceae, according to the first phylogenetic tree, which focuses on the core Xylariaceae (Figure 1). The second phylogenetic tree focuses on the Xylariales genera *incertae sedis*, and CMUB 40018, CMUB 40019, MFLU 18-0804, MFLU 19-2144, MFLU 23-0409, and MFLU 23-0410 form distinct clades in *Nigropunctata* and *Xenoanthostomella* (Figure 2).

### 3.2. Taxonomy

#### 3.2.1. Gyrotrichaceae Hern.-Restr. & Crous, in Hernández-Restrepo et al., *Persoonia* 49: 111 (2022) [35]

MycoBank: MB845985.

Notes: Hernández-Restrepo et al. [35] introduced the Gyrothricaceae into Xylariales to accommodate genera *Gyrothrix*, *Neogyrothrix*, *Pseudoceratocladium*, *Pseudocircinotrichum*, and *Xenoanthostomella*. The family is characterized by having gyrothrix- and circinotrichum-like asexual morphs and anthostomella-like sexual morphs.

*Xenoanthostomella* Mapook & K.D. Hyde, in Hyde et al., Fungal Divers. 100: 235 (2020) [55].

MycoBank: MB558737.

Notes: *Xenoanthostomella* was introduced by Hyde et al. [55] with the type *X*. *chromolaenae* on *Chromolaena odorata* from Thailand and accepted in Xylariales, *genera incertae sedis*, due to phylogenetic uncertainty. The genus has immersed ascomata beneath the clypeus, cylindrical to broadly filiform asci, and broadly fusiform, aseptate ascospores. Even though *Xenoanthostomella* is morphologically similar to the species of “*Anthostomella helicofissa*,” Samarakoon et al. [8] treated it as a separate genus until further studies were undertaken. Based on phylogeny and sexual morphology, Hernández-Restrepo et al. [35] discovered two Malaysian strains associated with *Albizia falcataria* petioles and *Falacia moluccana* seed pods that were connected to *X*. *chromolaenae*. In addition, *Xenoanthostomella* was accommodated into a family, the Gyrotrichaceae [35]. Currently, four *Xenoanthostomella* species are accepted: *X*. *calami* [84], *X*. *chromolaenae* [55], *X*. *cycadis* (≡ *Circinotrichum cycadis*), and *X*. *olivacea* (≡ *Helicotrichum olivaceum*) [35].

**Figure 3:**
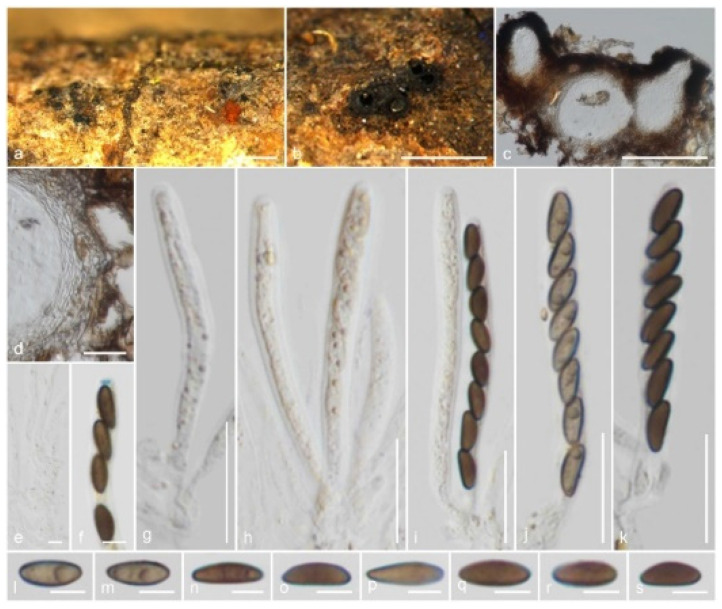
*Xenoanthostomella parvispora* (CMUB 40019, holotype): (**a**,**b**) Ascomata on the host surface; (**c**) Vertical section of ascomata; (**d**) Peridium; (**e**) Paraphyses; (**f**) Apical ring bluing in Melzer’s reagent; (**g**–**k**) Asci; (**l**–**s**) Ascospores. Scale bars: (**a**,**b**) = 500 μm; (**c**) = 100 μm; (**d**,**g**–**k**) = 20 μm; (**e**,**f**,**l**–**s**) = 5 μm.

*Xenoanthostomella parvispora* Samarak., sp. nov. (Figure 3).

MycoBank: MB850550.

Etymology: The specific epithet refers to the small ascospores.

Holotype: CMUB 40019.

Saprobic on the bark of a dead branch. Sexual morph: *Ascomata* 110–180 μm high, 70–110 μm diam., immersed, beneath clypeus, erumpent, visible as blackened, uni- or multilocular, in cross-section globose to obpyriform, coriaceous, solitary to scattered. *Clypeus* extending outwards around the ascomata, comprising dark fungal hyphae and host epidermal cells, thicker around the papilla. *Ostioles* centric or eccentric, ostiolar canal periphysate. *Peridium* 9–16 μm ($\bar{x}$ = 12.5 μm, *n* = 10) wide, thinner between two ascomata, with two cell layers: outer layer thick, comprising yellowish brown, thick-walled cells of *textura angularis*, and inner layer thin, composed of hyaline, thin-walled cells of *textura angularis*. *Paraphyses* 2–4 μm ($\bar{x}$ = 2.8 μm, *n* = 20) diam., intermingled among asci, prominent, hyphae-like, hyaline, smooth, septate, branched, thin-walled, guttulate, apically blunt. *Asci* 50–70 × 5–7 μm ($\bar{x}$ = 61.5 × 6.1 μm, *n* = 25), 8-spored, unitunicate, cylindrical, short-pedicellate, apically rounded with a 1.7–2.3 μm high, 0.6–1.5 μm diam., discoid apical ring, J+ in Melzer’s reagent. *Ascospores* 7.5–11 × 2.8–4.5 μm ($\bar{x}$ = 9.2 × 3.6 μm, *n* = 30), L/W 2.5, uniseriate, brown, inequilaterally ellipsoidal, aseptate, 1–2-guttulate, lacking a germ slit and appendage. Asexual morph: Undetermined.

Material examined: Thailand, Lamphun Province, on the bark of a dead branch of an unidentified dicotyledonous tree, 22 October 2022, M.C. Samarakoon, MC22-021 (CMUB 40019, holotype), (MFLU 23-0409, isotype). Additional sequences: CMUB 40019: OR507134 (*SSU*), OR504425 (*TEF-1α*).

Notes: *Xenoanthostomella parvispora* is described on the bark of a dead branch of an unidentified dicotyledonous tree from northern Thailand, which possesses generic characters such as immersed ascomata beneath a rudimentary clypeus, cylindrical to broadly filiform asci with a J+ apical ring, and broadly fusiform, aseptate ascospores. *Xenoanthostomella parvispora* differs from *X*. *chromolaenae* in having smaller ascomata (110–180 μm high, 70–110 μm diam. vs. 190–220 µm high, 175–210 µm diam.) and smaller ascospores (9.2 × 3.6 µm vs. 12 × 5 µm). Compared to *X*. *calami* and *X*. *chromolaenae*, *X*. *parvispora* lacks a germ slit. However, *X*. *cycadis* and *X*. *olivacea* are only known from their asexual morphs, thus morphological comparisons are not possible. The *ITS*, *LSU*, and *RPB2* sequences of *X*. *parvispora* are similar to those of *X*. *olivacea* CBS 101,185 (99%, 1/533 gaps; 99%, 0/871 gaps; 98%, 0/647 gaps), *X*. *cycadis* CBS 137,969 (97%, 7/550 gaps; 99%, 0/887 gaps; 91%, 0/720 gaps), and *X*. *chromolaenae* MFLU 18-0840 (88%, 27/570 gaps; 98%, 2/887 gaps; N/A). In our *ITS*, *LSU*, *RPB2*, and *TUB2* phylogeny (Figure 2), two of the isolates (CMUB 40,019 and MFLU 23-0409) form a monophyletic group sister to *X*. *olivacea* CBS 101,185 with strong statistical support (100% ML/1.00 PP). Here, we introduce *X*. *parvispora* as a new *Xenoanthostomella* species from Thailand.

#### 3.2.2. Xylariaceae Tul. & C. Tul. [as ‘Xylariei’], *Select. fung. carpol*. (Paris) 2: 3 (1863)

MycoBank: MB81528.

Notes: Xylariaceae is a well-established family in Xylariales including both conspicuous and inconspicuous forms of xylarialean taxa. Following several recent studies, Hyde et al. [9] accepted 32 genera in the Xylariaceae. However, with recent consecutive studies, several genera previously accepted in genera *incertae sedis* or uncertain taxonomy have been placed in the Xylariaceae based on morpho-molecular studies.

*Muscodor* Worapong, Strobel & W.M. Hess, Mycotaxon 79: 71 (2001).

MycoBank: MB28513.

Notes: *Muscodor* was introduced by Worapong et al. [60] based on characteristic volatile organic compounds (VOCs) profiles, *SSU* and *ITS* phylogeny, and hyphal morphologies such as coiling, ropiness, and branching patterns. The genus is typified by *M*. *albus* from the small limbs of *Cinnamomum zeylanicum* from Honduras. While numerous investigations have been carried out over the past two decades to identify novel *Muscodor* species as sterile mycelia and their VOCs, neither the sexual nor asexual morphs of the genus were recorded until 2020. Australia, Bolivia, Ecuador, India, and Thailand are among the tropical countries where a majority of *Muscodor* species have been described (e.g., [67,69,70,74,75]). In 2020, Samarakoon et al. [12] described two sexual morphic species that were closely related to *Muscodor* species phylogenetically. These two new taxa shared characteristics with the previously reported genus *Induratia*, such as apiosporous ascospores and inconspicuous ascomata forms. However, the herbarium specimen and molecular data of the generic type, *I*. *apiospora* (PDD 44399), were unavailable. Meanwhile, an *Induratia* sp. (SMH 1255) specimen originating from Puerto Rico [71] showed a close affinity to the above-mentioned two new sexual morphs. While considering the morphology of *I*. *apiospora* (PDD 44399; iconotype) and the morpho-molecular characteristics of *Induratia* sp. (SMH 1255), Samarakoon et al. [12] synonymized *Muscodor* into *Induratia* and introduced those two sexual morphic taxa as new species, *I*. *thailandica* (MFLU 18-0784; MFLUCC 17-2669) and *I*. *ziziphi* (MFLU 18-0107; MFLUCC 17-2662), and a new family, the Induratiaceae. Recently, Cedeño-Sanchez et al. [52] encountered an unpublished ex-holotype strain of *I*. *apiospora* among the holdings of the ATCC collection and conducted a detailed morpho-molecular study. The results showed that *I*. *apiospora* (ATCC 60639) clustered in the Barrmaeliaceae, and *Muscodor* was resurrected within the Xylariaceae. After recent crucial revisions of the genus, *Muscodor* accommodates 25 species (http://www.indexfungorum.org, accessed on 20 October 2023) and is accepted in the Xylariaceae. In this study, we introduce two new *Muscodor* species and an additional collection of *M*. *coffeanus*.

**Figure 4:**
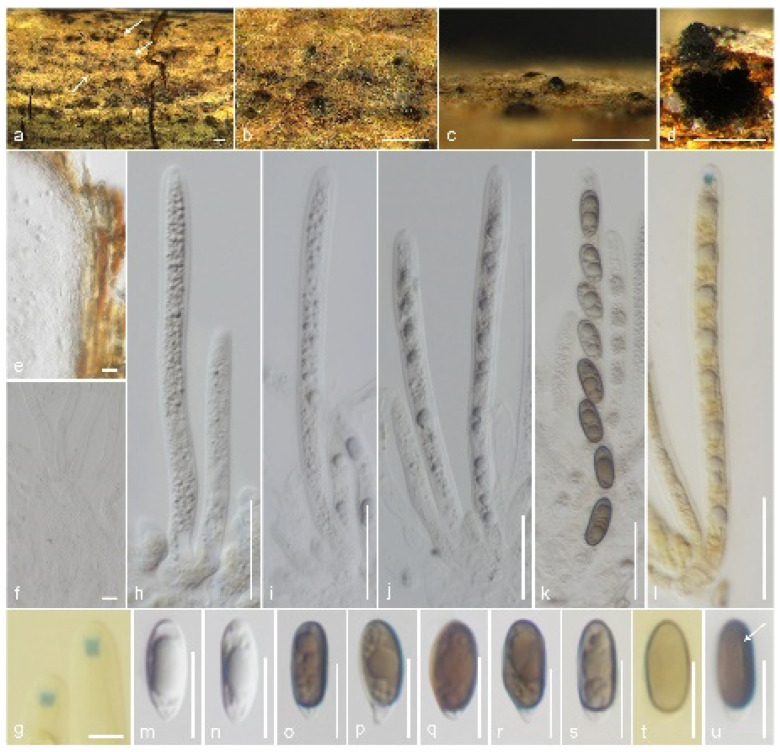
*Muscodor brunneascosporus* (CMUB 40020, holotype): (**a**–**c**) Ascomata on the host surface (ascomata shown in white arrows); (**d**) Vertical section of ascoma; (**e**) Section of peridium; (**f**) Paraphyses; (**g**) Apical ring bluing in Melzer’s reagent; (**h**–**l**) Asci ((**l**) in Melzer’s reagent); (**m**–**u**) Ascospores; (**t**) in Melzer’s reagent; (**u**) germ slit shows in a white arrow. Scale bars: (**a**–**c**) = 500 µm; (**d**) = 200 µm; (**h**–**l**) = 20 µm; (**e**,**m**–**u**) = 10 µm; (**f**,**g**) = 5 µm.

*Muscodor brunneascosporus* Samarak., sp. nov. (Figure 4).

MycoBank number: MB850551.

Etymology: The specific epithet refers to the Latin word that means ‘‘brown ascospores’.

Holotype: CMUB 40020.

Saprobic on a dead branch. Sexual morph: *Ascomata* 220–250 μm high, 225–325 μm diam., immersed, raised, visible as blackened, carbonaceous, solitary, in cross-section globose to sub-globose, tightly attached to substrate with a broad base. *Clypeus* black, thick-walled, short, comprising dark fungal hyphae and host epidermal cells. *Ostioles* raised from the center of ascomata. *Peridium* 11.5–16.5 μm ($\bar{x}$ = 13.8 μm, *n* = 12) thick, composed of two layers, occasionally not distinguished; inner layer hyaline, thin walled, 3–4 cell layers of *textura angularis*; outer layer yellowish brown, thick-walled, 4–6 cell layers of *textura angularis*. *Paraphyses* 3–5.5 μm ($\bar{x}$ = 4.2 μm, *n* = 30) diam., intermingled among asci, prominent, hyphae-like, hyaline, smooth, septate, thin-walled, guttulate, apically blunt. *Asci* 85–105 × 5.5–7.5 μm ($\bar{x}$ = 95 × 6.7 μm, *n* = 25), 8-spored, unitunicate, cylindrical, short-pedicellate, apically rounded with a 2–2.5 μm high, 1.1–1.6 μm diam., cylindrical, apical ring, J+ in Melzer’s reagent. *Ascospores* 13–16.5 × 4–6 μm ($\bar{x}$ = 14.5 × 5.3 μm, *n* = 30), L/W 2.7, uniseriate, ellipsoidal, constricted apiosporous; basal cell 1.5–3 μm ($\bar{x}$ = 2.2 μm, *n* = 30) length, hyaline, conical shape, guttulate; apical cell 10.5–12.5 μm ($\bar{x}$ = 11.4 μm, *n* = 30) length, brown, guttulate with a short germ slit, lacking an appendage. Asexual morph: Undetermined.

Material examined: Thailand, Lamphun Province, Ban Thi district, on a dead branch of an unidentified dicotyledonous tree, 24 September 2022, M.C. Samarakoon, MC22-011 (CMUB 40020, holotype), (MFLU 23-0406, isotype). Additional sequences: CMUB 40020: OR507135 (*SSU*), OR504426 (*TEF-1α*).

Notes: *Muscodor brunneascosporus* has inconspicuous stromatic forms with a rudimentary clypeus, 8-spored, unitunicate asci with a J+ apical ring, and uniseriate, ellipsoidal, constricted apiosporous ascospores. However, *M*. *brunneascosporus* differs from all the known sexual morphic *Muscodor* species in having apiosporous ascospores with hyaline basal cells and brown apical cells with a short germ slit. Similar ascospores can be observed among the inconspicuous xylarialeans in Xylariales genera *incertae sedis*. *Anthostomella sabiniana* is similar to *M*. *brunneascosporus* in having immersed ascomata (250 μm high, 240 μm diam. vs. 220–250 μm high, 225–325 μm diam.), filamentous paraphyses (4 μm vs. 4.2 μm wide), and apiosporous brown ascospores (13.5–16 × 5–7 μm vs. 13–16.5 × 4–6 μm). However, *A*. *sabiniana* differs from *M*. *brunneascosporus* in having larger asci (105–150 × 9–10 μm vs. 85–105 × 5.5–7.5 μm), a wedge-shaped apical ring, and a germ slit extending the full length of the basal brown cell [86]. *Brunneiapiospora* species are similar to *M*. *brunneascosporus* in having immersed ascomata under a clypeus, numerous paraphyses, and apiosporous ascospores with large brown apical cells [87]. However, none of the species is similar to *M*. *brunneascosporus* based on the combined morphological characters. The *LSU* sequence of *M*. *brunneascosporus* (CMUB 40020) is similar to that of *M*. *yunnanensis* W-S-38 (97%, 6/1193 gaps), *M*. *thailandica* HKAS 102,323 (96%, 7/1217 gaps), and *M*. *coffeanum* MFLUCC 13-0159 (96%, 5/1125 gaps). Isolates CMUB 40020 and MFLU 23-0406 form a clade with close affinity to *M*. *musae* JCM 18230 and *M*. *roseus* MONT 2098 (Figure 1). However, the species segregation among the taxa in a multigene phylogeny is not distinct. Since we failed to obtain cultures of these collections, the comparison of hyphal and culture morphologies is not possible. Based on the available data, we introduce *M*. *brunneascosporus* as a new species from Thailand.

**Figure 5:**
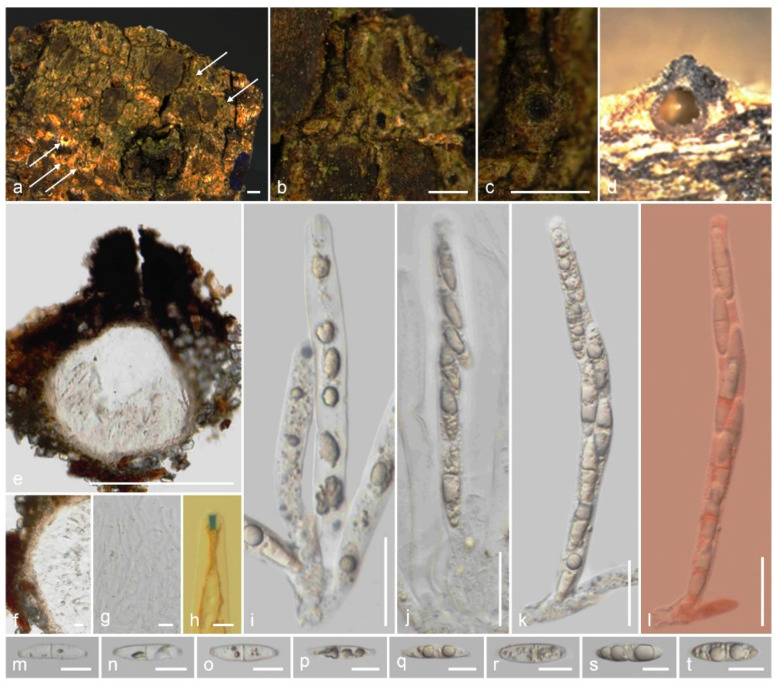
*Muscodor coffeanus* (CMUB 40022): (**a**–**c**) Ascomata on the host surface (ascomata shown in white arrows); (**d**,**e**) Vertical sections of ascomata; (**f**) Section of peridium; (**g**) Paraphyses; (**h**) Apical ring bluing in Melzer’s reagent; (**i**–**l**) Asci (**l** in Congo Red); (**m**–**t**) Ascospores. Scale bars: (**a**–**c**) = 500 µm; (**e**) = 200 µm; (**i**–**l**) = 20 µm; (**f**,**g**,**m**–**t**) = 10 µm; (**h**) = 5 µm.

*Muscodor coffeanus* A.A.M. Gomes, Pinho & O.L. Pereira [as ‘coffeanum’], in Hongsanan et al., *Cryptog. Mycol.* 36: 368 (2015) (Figure 5).

MycoBank: MB836101.

Saprobic on the bark of a dead branch. Sexual morph: *Ascomata* 275–350 μm high, 190–245 μm diam., semi-immersed, raised, visible as blackened, carbonaceous, solitary, in cross-section globose to sub-globose, tightly attached to substrate. *Clypeus* black, thick-walled, short, comprising dark fungal hyphae and host epidermal cells. *Ostioles* raised from the center of ascomata. *Peridium* 17.5–22 μm ($\bar{x}$ = 19.6 μm, *n* = 8) wide, composed of two layers; inner layer hyaline, thin-walled, 3–4 cell layers of *textura angularis*; outer layer yellowish brown, thick-walled, 3–4 cell layers of *textura angularis*. *Paraphyses* 2.5–5 μm ($\bar{x}$ = 3.6 μm, *n* = 30) diam., intermingled among asci, prominent, hyphae-like, hyaline, smooth, septate, thin-walled, guttulate, apically blunt, at times breaking into segments. *Asci* 90–125 × 8–9.5 μm ($\bar{x}$ = 111.5 × 8.7 μm, *n* = 25), 8-spored, unitunicate, cylindrical, short-pedicellate, apically rounded with a 3.4–4 μm high, 1.5–1.7 μm diam., cylindrical, apical ring, J+ in Melzer’s reagent. *Ascospores* 18–25 × 5–6.5 μm ($\bar{x}$ = 22.5 × 5.8 μm, *n* = 40), L/W 3.9, overlapping uniseriate, hyaline, bicellular, fusiform, ellipsoid-equilateral, with one median slightly constricted septum, lack constriction when immature, with rounded ends, guttulate, with polar cap-like sheaths, lacking a germ slit and appendage. Asexual morph: Undetermined.

Material examined: Thailand, Lamphun Province, on the bark of a dead branch of an unidentified dicotyledonous tree, 22 October 2022, M.C. Samarakoon, MC22-018 (CMUB 40022, MFLU 23-0407). Additional sequence: CMUB 40022: OR507136 (*SSU*).

Notes: *Muscodor coffeanus* was introduced by Hongsanan et al. [64] as an endophyte in stems of *Coffea arabica* from Brazil. The preliminary studies showed that the VOCs produced by *M*. *coffeanus* completely inhibited the growth of *Aspergillus ochraceous*, *A*. *niger*, *A*. *flavus*, and *Fusarium coffeanus* on PDA. Hyphal morphologies and *ITS* gene phylogeny have been used to introduce this species. Li and Kang [65] described the first report of the sexual morph of *M*. *coffeanus*, which was collected on the dead wood of an unknown plant from Chiang Mai. Our collection is morphologically similar to the *M*. *coffeanus* (MFLU 12-2129) sexual morph with slight differences in the ascomata and length of asci. MFLU 12-2129 has larger ascomata (380–450 μm high, 580–650 μm diam. vs. 275–350 μm high, 190–245 μm diam.) and longer asci (140–320 × 7–11.5 µm vs. 90–125 × 8–9.5 μm) compared to CMUB 40022. The ascomata (see Figure 2a,b in [65]) size and shape differences might be influenced by the texture of the substrate, as ascomata in our specimen were found immersed in the thick bark while those of Li and Kang [65] were in the soft xylem tissues. However, the size of 2-celled ascospores with equal divisions is in the overlapping range (18.5–22.5 × 6–8 µm vs. 18–25 × 5–6.5 μm). The *ITS* sequence of CMUB 40022 is similar to that of *M*. *yucatanensis* isolate 43 (99%, 0/512 gaps), *M*. *coffeanum* CDA739 (99%, 0/511 gaps), *M*. *coffeanum* MFLUCC 13-0159 (100%, 0/501 gaps), and *M*. *coffeanum* CDA743 (99%, 0/511 gaps), while the *LSU* sequence is similar to that of *M*. *coffeanum* MFLUCC 13-0159 (100%, 0/1114 gaps). Combined gene phylogenies also show that our new collection clusters in the *M*. *coffeanum* clade with strong statistical support sister to *M*. *coffeanum* MFLUCC 13-0159. Based on the similar morphologies and geography coupled with molecular analysis (Figure 1), our new material is added to *M*. *coffeanum* as an additional collection.

**Figure 6:**
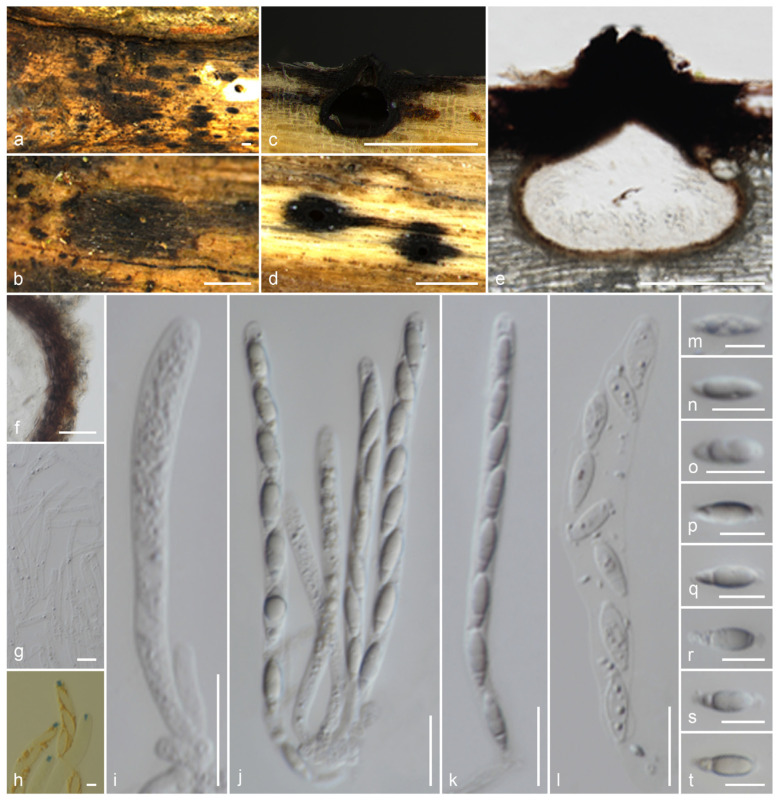
*Muscodor lamphunensis* (CMUB 40021, holotype): (**a**,**b**) Ascomata on the host surface; (**c**,**e**) Vertical sections of ascomata; (**d**) Longitudinal section of ascomata; (**f**) Section of peridium; (**g**) Paraphyses; (**h**) Apical ring bluing in Melzer’s reagent; (**i**–**l**) Asci; (**m**–**t**) Ascospores. Scale bars: (**a**–**d**) = 500 µm; (**e**) = 200 µm; (**f**,**i**–**l**) = 20 µm; (**g**,**m**–**t**) = 10 µm; (**h**) = 5 µm.

*Muscodor lamphunensis* Samarak., sp. nov. (Figure 6).

MycoBank number: MB850552.

Etymology: The specific epithet refers to Lamphun province, where the species was first collected.

Holotype: CMUB 40021.

Saprobic on a decorticated dead branch. Sexual morph: *Ascomata* 345–420 μm high, 365–410 μm diam., immersed, raised, visible as blackened, carbonaceous, solitary, in cross-section globose to sub-globose, tightly attached to substrate with a broad base. *Clypeus* black, thick-walled, short, comprising dark fungal hyphae and host epidermal cells. *Ostioles* raised from the center of ascomata. *Peridium* 16.5–35 μm ($\bar{x}$ = 24.6 μm, *n* = 10) wide, composed of two layers, occasionally not distinguished; inner layer hyaline, thin-walled, 3–4 cell layers of *textura angularis*; outer layer reddish brown, thick-walled, 6–8 cell layers of textura angularis. *Paraphyses* 2.5–4.5 μm ($\bar{x}$ = 3.6 μm, *n* = 30) diam., intermingled among asci, prominent, hyphae-like, hyaline, smooth, septate, thin-walled, guttulate, apically blunt. *Asci* 75–130 × 5.5–8.5 μm ($\bar{x}$ = 100.5 × 7 μm, *n* = 25), 8-spored, unitunicate, cylindrical, short-pedicellate, apically rounded with a 2.5–3.4 μm high, 1.7–2.7 μm diam., cylindrical, apical ring, J+ in Melzer’s reagent. *Ascospores* 13–17 × 3.8–5.8 μm ($\bar{x}$ = 15.1 × 5 μm, *n* = 35), L/W 3.02, uniseriate, ellipsoidal, mostly hyaline, constricted apiosporous; basal cell 2.5–6 μm ($\bar{x}$ = 4.3 μm, *n* = 30) length, conical shape, guttulate with remnant at the top; apical cell 9.5–12.7 μm ($\bar{x}$ = 11 μm, *n* = 30) length, rarely brown, guttulate with remnant at the base, lacking a germ slit and appendage. Asexual morph: Undetermined.

Material examined: Thailand, Lamphun Province, Ban Thi district, on a decorticated dead branch of an unidentified dicotyledonous tree, 24 September 2022, M.C. Samarakoon, MC22-008 (CMUB 40021, holotype), (MFLU 23-0408, isotype). Additional sequences: CMUB 40021: OR507137 (*SSU*), OR504427 (*TEF-1α*).

Notes: *Muscodor lamphunensis* is similar to *M*. *thailandica* (MFLU 18-0784) and *M*. *ziziphi* (MFLU 18-0107) in having inconspicuous stromatic forms with rudimentary clypeus, 8-spored, unitunicate, cylindrical asci with a J+ apical ring, and uniseriate, naviculate to ellipsoidal, mostly hyaline, smooth-walled constricted apiosporous ascospores with remnant at both polars [12]. *Muscodor lamphunensis* has larger ascomata and thick peridium compared to *M*. *thailandica* and *M*. *ziziphi*. On the substrate, we can observe a remarkably distributed black clypeus around the neck area in our collection. *Muscodor lamphunensis* and *M*. *ziziphi* share Type 1 paraphyses, as described by Samuels et al. [88]. The *ITS* sequence of *M*. *lamphunensis* CMUB 40021 is similar to that of *M*. *musae* CMU MU3 (99%, 1/601 gaps) and *M*. *albus* MONT 620 (99%, 1/601 gaps), while the *LSU* sequence is similar to that of *M*. *yunnanensis* W-S-38 (97%, 6/1193 gaps), *M*. *thailandica* HKAS 102,323 (96%, 6/1200 gaps), and *M*. *coffeanum* MFLUCC 13-0159 (96%, 5/1125 gaps). Few *RPB2* and *TUB2* sequences are publicly available, and the species have not been identified among some of them. Therefore, the sequence comparisons are incomplete for the related taxa. The sequence comparisons of the *RPB2* sequences from our new collection and the previously described three collections show significant differences as: *M*. *thailandica* MFLUCC 17-2669 (84%, 22/1086 gaps), *M*. *ziziphi* MFLUCC 17-2662 (83%, 22/1028 gaps), and *M*. *coffeanum* MFLUCC 13-0159 (85%, 16/1070 gaps). In our multigene phylogeny, two collections of *M*. *lamphunensis* (CMUB 40021 and MFLU 23-0408) cluster with *M*. *musae* JCM 18230, *M*. *oryzae* JCM 18231, and *M*. *roseus* MONT 2098 (Figure 1). However, the species segregation is not clear, probably due to the lack of other gene regions. There are no sexual or asexual morphologies of any species in this cluster. Since we failed to obtain a culture of this collection, the hyphal and culture morphologies are not possible. Even though *M*. *musae* JCM 18230 and *M*. *oryzae* JCM 18231 have been described from the same region, there is insufficient data to make any more comparisons. Based on the available morpho-molecular analyses, we introduce our new collection as a new species, *M*. *lamphunensis* from Thailand. However, it would not be surprising to find other species with asexual and sexual relationships, multigene phylogenies, and variable VOC profiles in the future, and to lump them together.

#### 3.2.3. Xylariales, *Genera Incertae Sedis*

*Nigropunctata* Samarak. & K.D. Hyde, in Samarakoon et al. *Fungal Divers*. 112: 68 (2022) [8].

MycoBank: MB558737.

Notes: *Nigropunctata* was introduced by Samarakoon et al. [8] to accommodate three new species, including the type species, *N*. *bambusicola*. *Nigropunctata* species are characterized by immersed ascomata; white or yellow ectostroma; cylindrical, short pedicel, apically rounded asci with J+, discoid or inverted hat-shaped apical ring, and cylindrical to broadly ellipsoidal, aseptate ascospores. Sugita et al. [89] introduced the Spirodecosporaceae to accommodate *Spirodecospora*, which has similar ascomata and asci morphologies to *Nigropunctata*. However, *Spirodecospora* species are characterized by broadly ellipsoidal to fusoid, aseptate, brown, verruculose ascospores with spirally or almost straight linear ornamentation. Interestingly, species from both *Nigropunctata* and *Spirodecospora* are described from bamboo substrates only. In this study, we introduced two new *Nigropuntata* species from Thailand, which are also collected from dead bamboo branches.

**Figure 7:**
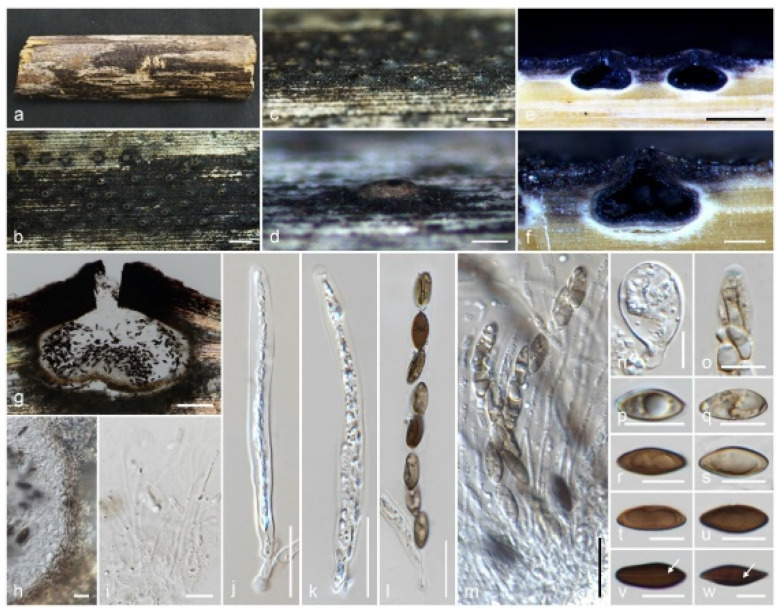
*Nigropunctata saccata* (MFLU 19-2144, holotype): (**a**) Substrate; (**b**–**d**) Ascomata on the host surface; (**e**–**g**) Vertical sections of ascomata; (**h**) Peridium; (**i**) Paraphyses; (**j**–**m**) Asci; (**n**) Sterile ascus like structure; (**o**) Apical ring bluing in Melzer’s reagent; (**p**–**w**) Ascospores (germ slits shown in white arrows). Scale bars: (**a**) = 1 cm; (**b**,**c**) = 1000 μm; (**e**) = 500 μm; (**d**,**f**) = 200 μm; (**g**) = 100 μm; (**j**–**m**) = 20 μm; (**h**,**i**,**n**–**w**) = 10 μm.

*Nigropunctata saccata* Samarak., sp. nov. (Figure 7).

MycoBank number: MB850553.

Etymology: The specific epithet refers to empty sac-like structures in ascomata.

Holotype: MFLU 19-2144.

Saprobic on a dead branch of bamboo. Sexual morph: *Ascomata* 290–370 μm high, 365–440 μm diam., immersed, dome-shaped areas, visible as black dots surrounded by round yellowish-brown patch, solitary or aggregated, in cross-section globose to sub-globose with an inwardly depressed base. *Clypeus* black, thick-walled, short, comprising dark fungal hyphae and host epidermal cells, flattened at the top. *Ostioles* centric or eccentric, periphysate ostiolar canal. *Peridium* 10–15 μm ($\bar{x}$ = 13 μm, *n* = 10) wide, yellowish white ectostroma, with two cell layers: outer layer thick, comprising brown, thick-walled cells of *textura angularis*, and inner layer thin, composed of hyaline, thin-walled cells of *textura angularis. Paraphyses* 2–3.3 μm ($\bar{x}$ = 2.6 μm, *n* = 15) diam., longer than the asci, numerous, hyaline, filamentous, sinuous, septate, constricted at septum, rarely branched, guttulate, blunt end *paraphyses* embedded in a gelatinous matrix. *Asci* 90–130 × 7–10 μm ($\bar{x}$ = 113 × 7.9 μm, *n* = 25), 8-spored, unitunicate, cylindrical, short pedicel, apically rounded with a discoid apical ring, J+ in Melzer’s reagent, often with broad sac like structures among asci. *Ascospores* 10.5–18 × 4.5–8 μm ($\bar{x}$ = 14.8 × 6.5 μm, *n* = 50), L/W 2.3, uniseriate or overlapping uniseriate, brown to dark brown, broadly ellipsoidal, aseptate, 1–2 guttules, germ slit on ventral side of the ascospore, straight, across the entire spore length. Asexual morph: Undetermined.

Material examined: Thailand, Chiang Rai, Amphoe Mueang district, on dead bamboo branches (Poaceae), 15 June 2019, M.C. Samarakoon, SAMC227 (MFLU 19-2144 holotype), (HKAS 107001, isotype). Chiang Mai Province, Amphoe Fang, on dead bamboo branches (Poaceae), 27 September 2016, M.C. Samarakoon, SAMC104 (MFLU 18-0804, HKAS 102304, paratype).

Notes: *Nigropunctata saccata* differs from other *Nigropunctata* species in having dome-shaped ostioles visible as black dots surrounded by a round yellowish-brown patch, a large clypeus, a white ectostroma, an inwardly depressed base, hyaline, globular sac-like structures among asci, a thin discoid apical ring, and a lack of mucilaginous sheath around ascospores. Our two collections share similar morphology, except MFLU 19-2144 possesses a thin peridium (10–15 μm), while MFLU 18-0804 possesses a thick peridium (18–23 μm). The *ITS* sequence of *N*. *saccata* is similar to that of *Anthostomella lamiacearum* MFLU 18-0101 (85%, 23/483 gaps), *Anthostomelloides leucospermi* CBS 110,126 (89%, 11/369 gaps), while the *LSU* sequence is similar to that of *Melanographium phoenicis* MFLUCC 18-1481 (94%, 4/857 gaps), *Xenoanthostomella chromolaenae* MFLUCC 17-1484 (93%, 10/854 gaps), *Haploanthostomella elaeidis* MFLU 20-0522 (93%, 18/857 gaps), *Gyrothrix encephalarti* CPC 35,966 (93%, 10/855 gaps), and *X*. *cycadis* CBS 137,969 (93%, 8/854 gaps). The *RPB2* sequence of *N*. *saccata* is similar to that of *N*. *bambusicola* MFLU 19-2145 (99%, 0/875 gaps), *N*. *thailandica* MFLU 19-2118 (94%, 0/873 gaps), and *Circinotrichum australiense* CBS 148,706 (80%, 8/724 gaps). In the multigene phylogeny, *N*. *saccata* forms a sister clade to *N*. *bambusicola* with medium statistical support (71% ML/0.98PP). Based on similar morphology and phylogeny, here we treat our new collection as a species of *Nigropunctata* and introduce a new species, *N*. *saccata*.

**Figure 8:**
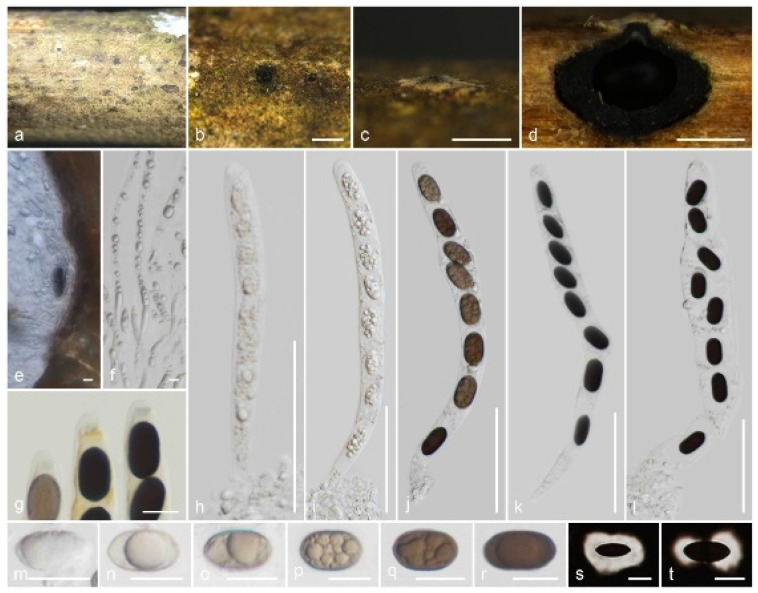
*Nigropunctata hydei* (CMUB 40018, holotype): (**a**) Substrate; (**b**,**c**) Ascomata on the host surface; (**d**) Vertical sections of ascoma; (**e**) Peridium; (**f**) Paraphyses; (**g**) Apical ring bluing in Melzer’s reagent; (**h**–**l**) Asci; (**m**–**t**) Ascospores (**s**,**t** in Indian ink). Scale bars: (**b**,**c**) = 500 μm; (**d**) = 200 μm; (**h**–**l**) = 50 μm; (**g**,**m**–**t**) = 10 μm; (**e**,**f**) = 5 μm.

*Nigropunctata hydei* Samarak., sp. nov. (Figure 8).

MycoBank number: MB850554.

Etymology: Named in honor of British mycologist Kevin D. Hyde, for his immense contributions to ascomycete taxonomy.

Holotype: CMUB 40018.

Saprobic on a dead branch of bamboo. Sexual morph: *Ascomata* 400–520 μm high, 485–575 μm diam., immersed, dome-shaped areas, visible as black dots surrounded by round yellowish-brown patches, solitary or aggregated, in cross-section globose to sub-globose with flattened bases. *Clypeus* black, thick-walled, short, comprising dark fungal hyphae and host epidermal cells, flattened at the top. *Ostioles* centric or eccentric, periphysate ostiolar canal. *Peridium* composed of two layers, occasionally not distinguished; 16.5–31 μm ($\bar{x}$ = 23.2 μm, *n* = 10) wide, yellowish-brown ectostroma, with two cell layers: outer layer thick, comprising brown, thick-walled cells of *textura angularis*, and inner layer thin, composed of hyaline, thin-walled cells of *textura angularis*. *Paraphyses* 3.5–5.6 μm ($\bar{x}$ = 4.6 μm, *n* = 30) diam., longer than the asci, numerous, hyaline, filamentous, sinuous, septate, constricted at septum, rarely branched, guttulate, blunt end paraphyses embedded in a gelatinous matrix. *Asci* 150–185 × 11.5–16.5 μm ($\bar{x}$ = 168.5 × 13.7 μm, *n* = 25), 8-spored, unitunicate, cylindrical, short-pedicellate, apically rounded with a 1.8–3.4 μm high, 3.8–5.2 μm diam., discoid apical ring, J+ in Melzer’s reagent. *Ascospores* 13.5–18 × 7–10 μm ($\bar{x}$ = 15.8 × 8.2 μm, *n* = 40), L/W 1.93, uniseriate or overlapping uniseriate, brown to dark brown, broadly ellipsoidal, aseptate, 1–2 guttules, covered with a thick, mucilaginous sheath, occasionally slightly constricted at the center, lacking a germ slit. Asexual morph: Undetermined.

Material examined: Thailand, Lamphun Province, on a dead branch of bamboo, 22 October 2022, M.C. Samarakoon, MC22-020 (CMUB 40018, holotype), (MFLU 23-0410, isotype). Additional sequence: CMUB 40018: OR507138 (*SSU*).

Notes: *Nigropunctata hydei* is similar to the other *Nigropunctata* species in having immersed ascomata with thick clypeus, ectostroma, cylindrical, short pedicel, apically rounded asci with J+, discoid apical ring, and broadly ellipsoidal, aseptate ascospores. As described in *N*. *saccata*, we noticed that the apical ring of *N*. *hydei* stained blue with Melzer’s reagent for only a short period. *Nigropunctata hydei* has larger ascomata (400–520 μm high, 485–575 μm diam.), wider peridium (16.5–31 μm wide), and larger asci (150–185 × 11.5–16.5 μm) compared to *N*. *bambusicola* (285–315 μm high, 260–340 μm diam., ascomata; 10–18 μm wide, peridium; 95–140 × 9.5–12.5 μm, asci) and *N*. *saccata* (290–370 μm high, 365–440 μm diam., ascomata; 10–15 μm wide, peridium; 90–130 × 7–10 μm, asci). *Nigropunctata hydei* differs from all *Nigropunctata* by lacking a germ slit. *Anthostomella zongluensis*, *A*. *lugubris*, and *A*. *tumulosa* share a similar size range of J+ apical ring and ellipsoidal, aseptate ascospores with a thick, mucilaginous sheath but lacking a germ slit. However, the ascomata and asci characters differ from those of *N*. *hydei* [86]. The *ITS*, *LSU*, and *RPB2* sequences of *N*. *hydei* are similar to those of *N*. *bambusicola* (92%, 12/558 gaps; 97%, 3/892 gaps; 91%, 0/963 gaps; 93%, 0/961 gaps) and *N*. *thailandica* (91%, 11/566 gaps; 97%, 0/955 gaps; 93%, 0/961 gaps). In the multigene phylogeny, *N*. *hydei* forms a sister clade to *N*. *thailandica* with medium statistical support. Here, we introduce *N*. *hydei*, a new species from Thailand based on morpho-molecular distinctions.

## 4. Discussion

New additions have occasionally altered the morpho-molecular taxonomy of the Xylariales/Amphisphaeriales/*incertae sedis*. Recent research on those *incertae sedis* taxa, which were previously known from a single collection, lacking recent collections, having inconsistent morphologies, no sexual–asexual links, and no molecular evidence, has urged the classification of genus to the family level. Families like the Appendicosporaceae [8], Barrmaeliaceae [32], Fasciatisporaceae [55], Gyrothricaceae [35], Oxydothidaceae [90], and Spirodecosporaceae [89] are some of the examples that have primarily been raised through studies on inconspicuous xylarialean taxa. Prior research has most likely focused on stromatic forms like diatrypoid, hypoxyloid, rosellinioid, and xylarioid rather than inconspicuous stromatic forms like anthostomelloid. The introduction of novel taxa does not adhere to these conservative approaches and emphasizes microscopic characteristics such as the type of ring, the color of the ascospores, and the presence or absence and type of germ slit [8].

*Muscodor*, *Nigropunctata*, and *Xenoanthostomella* are the three genera taxonomically identified during the study to focus on inconspicuous xylarialean taxa. If the stromatic nature was thought of as the key characteristic, those three genera would probably be treated as anthostomella-like taxa. Although *incertae sedis* taxa, particularly inconspicuous forms, are increasingly being split apart rather than being grouped because of molecular studies, we encountered several problems in each genus in their taxonomy and phylogeny.

With the addition of *Muscodor brunneascosporus* and *M*. *lamphunensis*, the number of species known from their sexual morphs has increased to five, which all are collected from northern Thailand [12,65]. There is a clear differentiation of the ascospore characters in those species. However, obtaining the asexual morphs from cultures has failed; therefore, the sexual–asexual connection is unknown. Due to the fact that many of the available sequences are only *ITS*, the species delineation based on phylogeny is uncertain. Stadler et al. [91] discussed the intragenomic polymorphism of the *ITS* region of the Hypoxylaceae and revealed less than 97%, or around 90%, of the overall homology of the *ITS* sequences. The intragenomic *ITS* variation among *Muscodor* species or anthostomella-like taxa needed to be checked to understand the *ITS* based species demarcation. *Nigropunctata* and *Spirodecospora* species are associated with dead bamboo and are similar to inconspicuous stromatic forms [89]. The microscopic morphologies and phylogenetic analyses support accepting them as distinct clades apart from the core Xylariaceae. This is important for the collection of fresh, inconspicuous xylarialean taxa and molecular phylogeny.

In summary, this study introduced five new xylarialean taxa (*Muscodor brunneascosporus*, *M*. *lamphunensis*, *Nigropunctata hydei*, *N*. *saccata*, and *Xenoanthostomella parvispora*), alongside the inclusion of *M*. *coffeanus* from northern Thailand. These species were all found on branches in varying stages of senescence and early decomposition during the rainy season. Nevertheless, the taxonomic classification of inconspicuous xylarialean taxa remains incomplete, and further investigations are needed, particularly with the inclusion of freshly collected specimens. The ongoing exploration of the taxonomy, phylogeny, and secondary metabolites of xylarialean taxa underscores northern Thailand’s significance as a focal point for continued research. Consequently, our future studies will be directed towards comprehensive investigations encompassing morphology, phylogeny, ecology, and the exploration of antimicrobial properties for potential applications in plant disease control.

## Data Availability

The DNA sequence data obtained from this study have been deposited in GenBank under accession numbers; *ITS* (OR507143–OR507151, MW240658, MW240663), *LSU* (OR507156–OR507164, MW240588, MW240593), *RPB2* (OR504419–OR504422, MW658642, MW658645), *SSU* (OR507134–OR507138), *TEF-1α* (OR504425–OR504427), and *TUB2* (OR519977–OR519979, MW775611, MW775613). The alignments are available in TreeBASE under the submission IDs 30764 and 30765.
